# Self‐Guided Psychological Interventions for the Treatment and Prevention of Eating Disorders: A Meta‐Analysis of Randomized Controlled Trials

**DOI:** 10.1002/erv.3201

**Published:** 2025-04-14

**Authors:** Jake Linardon, Claudia Liu, Zoe McClure, Hannah K. Jarman, Mariel Messer, Cleo Anderson, Matthew Fuller‐Tyszkiewicz

**Affiliations:** ^1^ SEED Lifespan Strategic Research Centre Faculty of Health School of Psychology Deakin University Geelong Australia; ^2^ Faculty of Health Office of the Executive Dean Deakin University Burwood Australia

**Keywords:** eating disorders, meta‐analysis, prevention, randomized controlled trial, self‐help, treatment

## Abstract

**Background:**

Self‐guided interventions may broaden the dissemination of evidence‐based prevention and treatment protocols for eating disorders. We conducted a meta‐analysis comparing self‐guided prevention and treatment approaches for eating disorders to (1) control groups and (2) professionally guided self‐help programs.

**Methods:**

Forty‐six trials were included. Interventions ranged from web, to app, to CD‐ROM to book‐based programs. Random effects meta‐analyses were conducted on numerous symptom and risk outcomes.

**Results:**

Only four trials recruited an unselected sample, with self‐guided programs reducing shape/weight concerns over control groups (*g* = 0.18). Among high risk/symptomatic samples (*k* = 25), self‐guided interventions reduced several behavioural and cognitive symptoms (*gs* = 0.31–0.50) over control groups, with effects being robust when adjusting for higher risk of bias and small sample trials. Among clinical samples (*k* = 17), evidence for the effectiveness of self‐guided interventions over control groups on symptom measures was only found for binge‐eating disorder, as too few studies sampled other diagnostic subtypes. Among 10 trials that compared guided to unguided self‐help, we observed a significant effect in symptom reduction in favour of guided self‐help (*g* = −0.26). Dropout did not differ between guided and unguided self‐help (OR = 0.97).

**Conclusion:**

Self‐guided interventions may be an effective, low intensity intervention format for high risk individuals or for binge‐eating disorder presentations.

**Summary**

- Conducted a meta‐analysis of 46 trials on self‐help interventions for eating disorders
- Self‐help interventions effectively reduced symptoms of eating disorders in pre‐selected and binge‐eating disorder samples.
- Guided self‐help produced a significantly larger effect size on symptom outcomes than pure self‐help.

## 1 Introduction

Eating disorders affect between 4% and 10% of the population (Hay et al. 2023), are characterised by chronicity and relapse, and are associated with an increased risk of mortality, suicide, depression, and substance abuse (Solmi et al. 2024). Despite the emergence of a range of evidence‐based prevention (Le et al. 2017) and treatment (Linardon et al. 2017) approaches, the reality is that fewer than one quarter of individuals with or at risk of an eating disorder have access to them (Weissman and Rosselli 2017). The reasons for this are complex, deep‐rooted and multi‐faceted, and typically include an insufficient number of adequately trained professionals, high costs of treatment, geographical isolation from available services, time constraints, perceived stigma associated with help‐seeking, and low motivation to change (Ali et al. 2017).

Self‐help interventions are a promising approach to circumvent existing help‐seeking barriers and close the treatment gap. Self‐help programs typically encompass a structured intervention or set of therapeutic skills that are based on a clear psychological model. Content is extracted from traditional therapist‐led manuals, and is simplified in a way that enables delivery through either a book, brochure, computer, the internet, or smartphone application (Yim and Schmidt 2019). Self‐help programs may be applicable across the entire continuum of care (from prevention, to early intervention, to treatment and relapse prevention), and can be administered with or without guidance from a professional. Guided self‐help involves provision of professional support that is primarily facilitatory in nature, designed to help the user address obstacles, clarify content, instil motivation, and review progress (Wilson and Zandberg 2012). Conversely, self‐guided approaches (also known as unguided self‐help) require the user to work through the programme independently, without any involvement from a professional (Cuijpers et al. 2011).

Self‐guided approaches offer unique advantages over professionally guided approaches. Their capacity to allow one to undergo treatment flexibly, autonomously, and at a preferred pace can help address the ambivalence toward help‐seeking and low motivation to change that are characteristic of eating disorders (Denison‐Day et al. 2018). Furthermore, since they do not require any input from a trained professional, their administration is not contingent upon the availability of providers, which means that multiple people can use a programme simultaneously, making them far more accessible, scalable and potentially cost‐effective (Edge et al. 2023).

A considerable number of self‐guided prevention, early intervention and treatment programs for eating disorders have been developed over the past 20 years. Most of these are based on cognitive‐behavioral therapy (CBT) manuals because there is a large evidence base supporting the efficacy of CBT (Linardon, Wade, et al. 2017). More recently, self‐guided programs that draw from diverse theoretical orientations and models of change have also emerged, including third‐wave behavioural approaches (e.g., dialectical behavioral therapy, compassion‐focused therapy, mindfulness‐based therapy), dissonance‐based interventions, and imagery rescripting.

Despite multiple randomized controlled trials (RCTs) evaluating the efficacy of self‐guided interventions, efforts to aggregate these trials, quantify the magnitude of their effects through meta‐analytic procedures, and understand the factors that may alter their efficacy are missing. Prior meta‐analyses on self‐help interventions have either focused exclusively on guided self‐help (Traviss‐Turner et al. 2017) or have amalgamated trials on both guided and unguided programs tested in specific formats (digitally‐delivered approaches; Linardon et al. 2020) or population groups (youth, people with a specific eating disorder subtype; Beintner et al. 2014; O'Mara et al. 2023). Broadly, these focused meta‐analyses have shown that a combination of guided and unguided self‐help programs produce small to moderate improvements in key eating disorder symptoms in the short‐term, with effects being tending to be stronger among trials sampling symptomatic participants and comparing the program to inactive controls (Linardon et al. 2020; Traviss‐Turner et al. 2017). Concerns have also been raised regarding the quality of evidence for self‐help interventions, as many initial trials had considerable risk of bias, affecting the interpretability of findings. Despite this, a comprehensive quantitative synthesis that locates all available trials on self‐guided programs delivered across the entire continuum of care for eating disorders is absent. Such a meta‐analysis is needed to more fully understand the utility of these inexpensive, low intensity intervention formats, as well as how and where they may be best situated in clinical care for eating disorders.

It is also unclear how self‐guided approaches fare in comparison to professionally guided approaches. Professionally guided approaches are thought to be more effective because they provide individuals with structured support, greater accountability, and tailored strategies that can enhance understanding of therapeutic concepts, making it easier to apply the core techniques in daily life (Cuijpers et al. 2010; Tong et al. 2024). While numerous trials comparing the two approaches among clinical and high risk eating disorder samples have been conducted over the past 10 years, conflicting results appeared to have emerged, with some trials reporting superiority of guided over unguided approaches (Kass et al. 2014) and other smaller trials failing to detect group differences (Carter et al. 2020). Because any differences between guided and unguided approaches are likely small in magnitude, it is possible that many of these studies were underpowered to detect significant group differences, resulting in biased estimates of between‐group differences. Aggregating trials that directly compare unguided to guided self‐help via meta‐analytic procedures can overcome the limitations of sample size in individual trials and provide more accurate estimates of effects that might guide further comparisons of these intervention formats in the future.

The aims of the present meta‐analysis were threefold: (1) to examine the efficacy of self‐guided interventions for the prevention, management and treatment of eating disorders relative to control groups; (2) to investigate whether major study characteristics moderate these efficacy estimates; and (3) to test the relative efficacy of self‐guided and professionally guided interventions among trials that performed a direct, head‐to‐head comparison.

## 2 Method

### 2.1 Study Selection

This review (pre‐registered at https://osf.io/gk3fr) was conducted in accordance to the Preferred Reporting Items for Systematic Reviews and Meta‐Analyses (PRISMA) guidelines (Page et al. 2021). The primary searched involved searching the Medline, PsycINFO, Web of Science and ProQuest Dissertations online databases (Oct 2024) by combining key words related to eating disorders, randomized controlled trials, and self‐help. The full list of search terms are presented in the Supplementary Materials. We also searched through prior reviews on eating disorder treatment to identify any study that may not have been captured through the primary search. No data restrictions were applied.

We included RCTs that tested the effects of a self‐guided prevention, risk factor reduction, or treatment programme for eating disorders against either a control condition or a guided version of the same programme. Both pilot and full‐scale trials were eligible for inclusion. Self‐guided interventions were defined as a structured programme or a prescribed set of therapeutic tasks that participants were instructed to work through independently without any guidance from a professional, researcher, or peer. Any support offered must either have been in reference to technical aspects of the study or intervention (e.g., troubleshooting technical difficulties), or support that was automated (e.g., standardized reminder emails) or provided by a computer (e.g., chat‐bots). The intervention could be delivered via a book, web platform, smartphone application, audio recordings, or computer. Guided self‐help comparisons were defined as structured programs or therapeutic tasks accompanied by regular professional support. This support was primarily facilitatory, aimed at helping users engage with content, clarify key concepts, and maintain motivation. Importantly, guided self‐help does not include therapist‐led programs where therapists direct the content and structure of sessions. For example, standard in‐person treatments adapted for technological delivery (e.g., via Skype) were excluded. Support could be provided through various means, including email, in‐person meetings, or phone calls. There were no sample restrictions, because we wanted to examine the effects of self‐help interventions as both prevention (delivered to universal or at risk samples) and treatment (delivered to clinically diagnosed samples) approaches. Published and unpublished trials were eligible.

Records from the databases were combined and duplicate records were removed. Titles and abstracts were screened to determine potential eligibility. The full‐text of these articles was then read to confirm whether full inclusion criteria were met. Authors of articles that did not provide sufficient data to calculate an effect size were contacted. Two researchers performed the study screening and selection methods.

### 2.2 Risk of Bias and Data Extraction

Four criteria from the Cochrane Collaboration Risk of Bias tool (Higgins and Green 2011) were used to assess risk of bias among the eligible studies. These criteria include random sequence generation, allocation concealment, blinding of outcome assessment, and incomplete outcome data. Each of the four domains were rated as either high risk, low risk or unclear. Selection bias was rated as low risk if there was a random component in the allocation sequence generation. Allocation concealment was rated as low risk when a clear method that prevented foreseeing group allocation before or during enrolment was stated. Blinding of outcome assessors was rated as low risk if proper measures were taken to conceal participants' group membership, or if the outcome measures were self‐reported, which does not involve direct contact with the researcher. Incomplete outcome data was rated as low if the trial authors conducted intention‐to‐treat analyses that included all randomized participants in the analyses.

We also extracted various characteristics related to the participants, intervention, control group, follow‐up length, sample size, and outcome assessments. Two independent researchers extracted information from eligible trials. Minor discrepancies were resolved through consensus.

### 2.3 Outcome Variables

Ten outcomes were subject to meta‐analysis given adequate reporting across the available trials. Eight outcomes were continuous and included general eating disorder psychopathology, objective binge eating, compensatory behaviours, shape/weight concerns, dietary restraint, eating concerns, and appearance internalisation. Two outcomes were binary and included abstinence (cessation of binge eating and/or purging) and dropout (failure to complete the follow‐up assessment^1^). We prioritised outcomes that were assessed via interview over self‐report if a trial reported use of both. The assessment tools used for each outcome are described in the Supporting Information S1.

### 2.4 Meta‐Analysis

For continuous outcomes, the effect size was calculated by dividing the difference between the group means at post‐test by the pooled standard deviation. Effect sizes were then reported as Hedges' *g* to correct for small sample bias (Hedges and Olkin 1985). If data from both intention‐to‐treat and completer analyses were presented, the former was extracted and analysed. If more than one measure of a particular outcome was reported, the mean of the effect sizes from each measure within the study was calculated, before the effect sizes were pooled (Lipsey and Wilson 2001). Effect sizes of 0.8 were interpreted as large, while effect sizes of 0.5 as moderate, and effect sizes of 0.2 as small (Cohen 1992). We also reported the number‐needed‐to‐treat (NNT), which indicates the number of additional participants in the intervention group who would need to be treated in order to observe one participant who shows positive symptom change relative to the control group (Cook and Sackett 1995).

For binary outcomes (abstinence and drop‐out), odds ratios (OR) were reported as the measure of effect. ORs were calculated separately for each study, weighted by their inverse variance, and then pooled to create a summary effect. ORs > 1 indicate that abstinence and dropout rates were higher in the self‐guided group than the control group.

We also conducted several sensitivity analyses to assess whether the main findings were robust. We re‐calculated the pooled effects when restricting the analyses to lower risk of bias trials (defined as meeting all of the quality criteria) and the smallest and largest effect in each study, if multiple conditions were used (to maintain statistical independence). We also pooled effects while excluding outliers using the non‐overlapping confidence interval (CI) approach, in which a study is defined as an outlier when the 95% CI of the effect size does not overlap with the 95% CI of the pooled effect size (Harrer et al. 2021). Small‐study bias was also examined through the trim‐and‐fill method (Duval and Tweedie 2000).

Since we expected considerable heterogeneity among the studies, random effects models were employed for all analyses. Heterogeneity was examined by calculating the *I* ^2^ statistic, which quantifies heterogeneity revealed by the Q statistic and reports how much overall variance (0%–100%) is attributed to between‐study variance (Higgins and Thompson 2002). We also conducted a series of subgroup analyses, examining the effects of the intervention according to major characteristics of the participants, intervention, and trial. Subgroup analyses were conducted under a mixed effects model. All analyses were conducted using Comprehensive Meta‐Analysis Version 3.0 (Borenstein et al. 2009).

## 3 Results

Supporting Information S1: Figure S1 presents a flowchart of the literature search. There were 46 trials (three unpublished) that met the full inclusion criteria. We present results from the meta‐analysis separately for unselected, pre‐selected, and clinical samples (defined below).

### 3.1 Self‐Guided Interventions Versus Controls

#### 3.1.1 Unselected Samples

*Characteristics*. Only four trials recruited an unselected sample, which were defined as whole populations for which participants were not screened to be at risk nor required to exhibit any symptoms. There were three CBT conditions, and one gratitude and mindfulness condition. Two trials delivered web interventions, while one trial each delivered an app and a self‐help book. Waitlists were used in two trials, while one trial used information resources and a placebo control book. Participant age ranged from 20 to 33 years, and sample size at post‐test ranged from 108 to 414. Only one trial (Wilksch et al. 2018) delivered an intervention explicitly designed for universal prevention; the others delivered a programme of therapeutic skills from established prevention and early intervention protocols to a sample that were not screened for eligibility. Intervention length ranged from 3 to 10 weeks and publication year ranged from 2017 to 2023. One trial met all four risk of bias criteria, one trial met two of the criteria, and two trials only met one criterion (see Supporting Information S1: Table S1).

*Efficacy Estimates*. Table 1 presents the results of the meta‐analyses comparing self‐guided interventions to control groups for unselected samples. Only three outcomes were available for analyses given the small number of trials recruiting an unselected sample. Significant, small pooled effect sizes in favour of self‐guided interventions over control conditions were observed for the five comparisons for shape/weight concerns (*g* = 0.18, 95% CI = 0.05, 0.31; *I* ^2^ = 0%), corresponding to a number‐needed‐to‐treat of 18.4. These effects remained robust in sensitivity analyses, including when restricting the analyses to one effect per study and low risk of bias trials, and when applying the trim‐and‐fill procedure. However, non‐significant effects were observed for the comparisons between self‐guided interventions and control conditions on eating disorder psychopathology (*g* = 0.03, 95% CI = −0.10, 0.16; *I* ^2^ = 0%) and internalisation (*g* = 0.11, 95% CI = −0.05, 0.27; *I* ^2^ = 0%).

**TABLE 1:** Meta‐analysis on the effects of self‐guided interventions versus control groups for unselected samples.

	Eating disorder psychopathology				Shape/Weight concerns				Internalisation			
Analysis	*N*_comp_	*g* (95% CI)	*I* ^2^	NNT	*N*_comp_	*g* (95% CI)	*I* ^2^	NNT	*N*_comp_	*g* (95% CI)	*I* ^2^	NNT
Self‐guided versus control												
Total effect	5	0.03 (−0.10, 0.16)	0%	117.5	5	0.18 (0.05, 0.31)*	0%	18.4	2	0.11 (−0.05, 0.27)	0%	31.0
One effect per trial (smallest)	3	−0.01 (−0.19, 0.16)	0%		3	0.19 (0.02, 0.35)*	0%	17.4	1	0.05 (−0.19, 0.29)	0%	69.9
One effect per trial (largest)	3	0.11 (−0.05, 0.29)	0%	31.0	3	0.21 (0.04, 0.37)*	0%	15.6	1	0.17 (0.05, 0.39)	0%	19.6
Low risk of bias trials	0	—	—		1	0.26 (0.01, 0.53)*	0%	12.4	0	—		
Trim‐and‐fill estimate	4	0.01 (−0.11, 0.14)		355.7	4	0.16 (0.04, 0.27)*	—	20.9				

*Note:* There were no outliers detected in these analyses.

Abbreviations: *N*_comp_ = number of comparisons; NNT = Number‐needed‐to‐treat; OR = Odds ratio.

* *p* < 0.05.

### 3.2 Pre‐Selected Samples

*Characteristics*. Twenty‐five trials recruited a pre‐selected sample, in which participants were deemed high risk or were symptomatic, but without being formally diagnosed. The pre‐selection criteria varied across the studies; seven trials screened purely for elevated weight concerns using the Weight Concerns Scale, six screened for the presence of behavioural symptoms (e.g., binge eating), five required participants to self‐identify body image problems or eating concerns, three screened for elevated perfectionism, and the rest screened for a mix of behavioural and cognitive symptoms. There were 30 intervention conditions; 17 were based on CBT, three on third‐wave approaches (i.e., acceptance and commitment therapy, mindfulness, and dialectical behavior therapy [DBT]), seven on dissonance interventions, two on psychoeducation with symptom monitoring, and one on imagery rescripting. A web programme was delivered most frequently (23 conditions), followed by an app (4 conditions), a self‐help book (2 conditions), and a chat‐bot (one condition). Nine of these trials delivered a programme that was designed for prevention, namely the *Body Project* and its variants or *Student Bodies.* The others were not clear whether prevention or treatment were the focus. Intervention length ranged from 1 to 20 weeks. Sample size at post‐test ranged from 21 to 373, and publication year ranged from 2005 to 2025. There were 32 comparison conditions, with a waitlist administered most often (20 conditions). Thirteen trials met all four quality criteria, three met three criteria, six two criteria, and two only met one criterion (Supporting Information S1: Table S1).

*Efficacy Estimates*. Six outcomes were available for analyses among pre‐selected samples. Significant, moderate pooled effect sizes in favour of self‐guided interventions over control groups were observed for eating disorder psychopathology (*g* = 0.42; 95% CI = 0.27, 0.56; NNT = 7.3; *I* ^2^ = 79%), binge eating (*g* = 0.47; 95% CI = 0.36, 0.58; NNT = 6.4; *I* ^2^ = 0%), shape/weight concerns (*g* = 0.41, 95% CI = 0.32, 0.50; NNT = 7.5; *I* ^2^ = 33%), and eating concerns (*g* = 0.50, 95% CI = 0.38, 0.62; NNT = 6.0; *I* ^2^ = 30%). Smaller but significant pooled effect sizes were also observed for compensatory behaviours (*g* = 0.29, 95% CI = 0.17, 0.41; NNT = 11.0; *I* ^2^ = 0%) and dietary restraint (*g* = 0.35, 95% CI = 0.22, 0.47; NNT = 8.9; *I* ^2^ = 56%). All effects remained significant and similar in magnitude across the various sensitivity analyses (Table 2).

**TABLE 2:** Meta‐analysis on the effects of self‐guided interventions versus control groups for pre‐selected samples.

	Eating disorder psychopathology				Binge eating				Compensatory behaviours			
Analysis	*N*_comp_	*g* (95% CI)	*I* ^2^	NNT	*N*_comp_	*g* (95% CI)	*I* ^2^	NNT	*N*_comp_	*g* (95% CI)	*I* ^2^	NNT
Self‐guided versus control												
Total effect	29	0.42 (0.27, 0.56)*	79%	7.3	6	0.47 (0.36, 0.58)*	0%	6.4	4	0.29 (0.17, 0.41)*	0%	11.0
One effect per trial (smallest)	22	0.41 (0.24, 0.59)*	82%	7.5	5	0.44 (0.32, 0.56)*	0%	6.9	3	0.25 (0.11, 0.38)*	0%	12.9
One effect per trial (largest)	22	0.46 (0.28, 0.63)*	82%	6.4	5	0.46 (0.34, 0.58)*	0%	6.6	3	0.29 (0.13, 0.40)*	0%	11.0
Outliers excluded	24	0.43 (0.33, 0.53)*	44%	7.1	6	0.47 (0.36, 0.58)*	0%	6.4	4	0.29 (0.17, 0.41)*	0%	11.0
Low risk of bias trials	17	0.45 (0.32, 0.57)*	62%	6.7	5	0.47 (0.36, 0.58)*	0%	6.4	4	0.29 (0.17, 0.41)*	0%	11.0
Trim‐and‐fill estimate	22	0.54 (0.39, 0.69)*	—	5.5	5	0.48 (0.38, 0.58)*		6.3	3	0.25 (0.14, 0.36)*		12.9

	Shape/Weight concerns				Eating concerns				Dietary restraint			
	*N*_comp_	*g* (95% CI)	*I* ^2^	NNT	*N*_comp_	*g* (95% CI)	*I* ^2^	NNT	*N*_comp_	*g* (95% CI)	*I* ^2^	NNT
Total effect	18	0.41 (0.32, 0.50) *	33%	7.5	7	0.50 (0.38, 0.62)*	30%	6.0	14	0.35 (0.22, 0.47)*	56%	8.9
One effect per trial (smallest)	14	0.39 (0.29, 0.49) *	35%	7.9	6	0.46 (0.36, 0.57)*	0%	6.6	11	0.31 (0.18, 0.43)*	50%	10.2
One effect per trial (largest)	14	0.43 (0.35, 0.52) *	17%	7.1	6	0.48 (0.34, 0.61)*	31%	6.3	11	0.33 (0.19, 0.46)*	56%	9.5
Outliers excluded	15	0.42 (0.33, 0.51)*	26%	7.3	7	0.50 (0.38, 0.62)*	30%	6.0	12	0.29 (0.17, 0.40)*	40%	11.0
Low risk of bias trials	9	0.48 (0.40, 0.57) *	0%	6.3	6	0.52 (0.42, 0.62)*	8%	5.7	8	0.43 (0.27, 0.58)*	62%	7.1
Trim‐and‐fill estimate	18	0.41 (0.32, 0.50) *		7.5	6	0.53 (0.39, 0.66)*		5.6	13	0.37 (0.24, 0.49)*		8.4

*Note:* Bold denotes statistical significance.

Abbreviations: *N*_comp_ = number of comparisons; NNT = Number‐needed‐to‐treat; OR = Odds ratio.

* *p* < 0.05.

### 3.3 Clinical Samples

*Characteristics*. Seventeen trials recruited a clinical sample, comprised either of bulimia nervosa (*k* = 6), binge‐eating disorder (*k* = 7), mixed diagnoses (*k* = 3), and anorexia nervosa (*k* = 1). There were 18 self‐help conditions (one trial delivered two self‐help programs); most were based on CBT (13 conditions) while others were compassion‐focused (2 conditions), DBT (1 condition), and psychoeducation (1 condition). Nine interventions were delivered via a book, four via the web, four via a computer, and one via handout sheets. Intervention length ranged from 2 to 24 weeks, and sample size at post‐test ranged from 35 to 129. Trials were published between 1998 and 2024. All trials delivered a programme pitched as an early treatment, whereas one delivered psychoeducational resources. The mean age of participants ranged from 25 to 40 years. There were 26 comparison conditions, with a waitlist being administered in 12 conditions. Six trials met all four quality criteria, one met three criteria, seven two criteria, and three only met one criterion (Supporting Information S1: Table S1).

*Efficacy Estimates*. Seven outcomes were available for meta‐analysis among clinical samples (Table 3). Significant, small‐medium pooled effect sizes favouring self‐guided interventions over control conditions were observed for eating disorder psychopathology (*g* = 0.40, 95% CI = 0.20, 0.60; NNT = 7.7; *I* ^2^ = 46%) and binge eating (*g* = 0.38, 95% CI = 0.22, 0.54; NNT = 8.1; *I* ^2^ = 0%). Results largely remained robust in sensitivity analyses and when restricting the analyses to specific diagnoses, although the number of studies was low. Abstinence from binge‐purge behaviours was significantly higher among those allocated to the self‐guided intervention over the control group (OR = 2.33, 95% CI = 1.24, 4.35; *I* ^2^ = 0%), although this effect was non‐significant when taking into account small study bias. Small, non‐significant effects were observed for compensatory behaviours, shape/weight concerns, eating concerns, and dietary restraint.

**TABLE 3:** Meta‐analysis on the effects of self‐guided interventions versus control groups for clinical samples.

	Eating disorder psychopathology				Binge eating				Compensatory behaviours			
Analysis	*N*_comp_	*g* (95% CI)	*I* ^2^	NNT	*N*_comp_	*g* (95% CI)	*I* ^2^	NNT	*N*_comp_	*g* (95% CI)	*I* ^2^	NNT
Self‐guided versus control												
Total effect	11	0.40 (0.20, 0.60)*	46%	7.7	10	0.38 (0.22, 0.54)*	0%	8.1	3	0.26 (−0.01, 0.53)	10%	12.4
Bulimia nervosa samples only	3	0.39 (−0.17, 0.97)	82%	7.9	3	0.34 (0.02, 0.66)*	32%	9.2	3	0.26 (−0.01, 0.53)	10%	12.4
Binge‐eating disorder samples only	5	0.56 (0.32, 0.80)*	0%	5.2	6	0.44 (0.22, 0.66)*	0%	6.9	0	—		
One effect per trial (smallest)	10	0.37 (0.17, 0.57)*	44%	8.4	9	0.36 (0.20, 0.53)*	0%	8.6	3	0.26 (−0.01, 0.53)	10%	12.4
One effect per trial (largest)	10	0.41 (0.20, 0.62)*	50%	7.5	9	0.37 (0.20, 0.54)*	5%	8.4	3	0.26 (−0.01, 0.53)	10%	12.4
Low risk of bias trials	5	0.36 (0.12, 0.59)*	41%	8.6	3	0.50 (0.27, 0.72)*	0%	6.0	1	0.36 (0.02, 0.71)*	0%	8.6
Trim‐and‐fill estimate	11	0.40 (0.20, 0.60)*		7.7	9	0.41 (0.24, 0.58)*		7.5	3	0.26 (−0.01, 0.53)	10%	12.4

	Shape/Weight concerns				Eating concerns				Dietary restraint			
	*N*_comp_	*g* (95% CI)	*I* ^2^	NNT	*N*_comp_	*g* (95% CI)	*I* ^2^	NNT	*N*_comp_	*g* (95% CI)	*I* ^2^	NNT
Total effect	6	0.20 (−0.23, 0.64)	77%	16.5	5	0.26 (−0.47, 1.01)	90%	12.4	5	0.17 (−0.18, 0.52)	61%	19.6
Bulimia nervosa samples only	4	0.01 (−0.56, 0.58)	82%	355.7	4	0.04 (−0.74, 0.83)	90%	87.8	4	0.12 (−0.31, 0.56)	69%	28.3
Binge‐eating disorder samples only	1	0.55 (0.03, 1.07)*	0%	5.4	1	1.19 (0.62, 1.73)*	0%	2.2	1	0.35 (−0.16, 0.86)	0%	8.9
One effect per trial (smallest)	5	0.31 (−0.14, 0.77)	75%	10.2	4	0.43 (−0.42, 1.30)	91%	7.1	4	0.24 (−0.16, 0.65)	65%	13.5
One effect per trial (largest)	5	0.32 (−0.12, 0.77)	73%	9.9	4	0.44 (−0.41, 1.30)	91%	6.9	4	0.26 (−0.12, 0.64)	61%	12.4
Low risk of bias trials	4	−0.06 (−0.43, 0.37)	66%		4	0.02 (−0.69, 0.73)	87%	177.1	4	0.01 (−0.23, 0.26)	0%	355.7
Trim‐and‐fill estimate	6	0.20 (−0.23, 0.64)		16.5	4	0.02 (−0.74, 0.79)		177.1	5	0.17 (−0.18, 0.52)		19.6

	Abstinence		
	*N*_comp_	OR (95% CI)	*I* ^2^
Total effect	7	2.33 (1.24, 4.35)*	0%
Bulimia nervosa samples only	2	1.64 (0.56, 4.75)	0%
Binge‐eating disorder samples only	3	3.26 (0.70, 15.10)	55%
Low risk of bias trials	2	3.08 (0.48, 19.54)	67%
Trim‐and‐fill estimate	4	1.67 (0.83, 3.36)	

*Note:* There were no outliers detected in these analyses. Bold denotes statistical significance.

Abbreviations: *N*_comp_ = number of comparisons; NNT = Number‐needed‐to‐treat; OR = Odds ratio.

* *p* < 0.05.

### 3.4 Subgroup Analyses

We conducted a series of subgroup analyses to determine whether various trial characteristics moderated effect sizes. Subgroup analyses were only conducted on the eating disorder psychopathology outcome given the low number of trials reporting the other outcomes. We combined trials on pre‐selected and clinical (but not unselected) samples for these subgroup analyses. We did not include the four trials on unselected samples because we deemed them to be vastly different to pre‐selected and clinical samples, so combining them would introduce too much heterogeneity and affect the interpretability of any subgroup effects. We analysed the following potential moderators: sample type (pre‐selected vs. clinical); CBT intervention (yes vs. no); dissonance‐based intervention (yes vs. no); third‐wave intervention (yes vs. no); delivery format (book vs. digital); control group (waitlist vs. other control); and follow‐up length (1–4 weeks vs. 5–8 weeks vs. 9+ weeks).

Two significant moderators emerged. Trials that delivered a dissonance‐based intervention (vs. other intervention types) produced significantly smaller effects, while trials that administered a waitlist control (vs. other control conditions, comprised of either generic self‐help books, informational resources, care as usual, or other types of placebos) produced significantly larger effects. Sample type, delivery of a CBT or third‐wave intervention, programme format, and follow‐up duration were not significantly associated with effect sizes (Table 4).

**TABLE 4:** Subgroup analyses combining pre‐selected and clinical sample trials on eating disorder psychopathology.

Subgroup	*N*_comp_	g (95% CI)	*I* ^2^	*p*
Sample type				0.887
Pre‐selected	29	0.42 (0.27, 0.56)	79%	
Clinically diagnosed	11	0.40 (0.20, 0.60)	46%	
CBT intervention				0.080
Yes	24	0.49 (0.32, 0.67)	78%	
No	16	0.29 (0.15, 0.43)	54%	
Third‐wave intervention				0.520
Yes	5	0.48 (0.31, 0.65)	0%	
No	35	0.41 (0.27, 0.54)	77%	
Dissonance‐based intervention				**0.001**
Yes	7	0.14 (0.03, 0.26)	0%	
No	33	0.46 (0.33, 0.60)	765	
Delivery format				0.221
Digital	34	0.37 (0.27, 0.48)	63%	
Book	6	0.88 (0.09, 1.66)	92%	
Control group				**0.001**
Waitlist	28	0.51 (0.35, 0.66)	77%	
Other	12	0.18 (0.07, 0.28)	7%	
Follow‐up				0.713
1–4 weeks	19	0.47 (0.35, 0.59)	41%	
5–8 weeks	10	0.45 (0.11, 0.78)	90%	
9 + weeks	10	0.39 (0.21, 0.56)	43%	

*Note:* Bold denotes statistical significance.

### 3.5 Unguided Versus Guided Self‐Help

Ten trials (12 comparisons) directly compared unguided to guided self‐help. Seven trials delivered a CBT programme, one a DBT programme, and two a psychoeducational programme with a symptom monitoring platform. Six trials sampled clinical populations (*k* = 2 transdiagnostic samples, *k* = 3 binge‐eating disorder samples, and *k* = 1 bulimia nervosa sample), while four recruited a pre‐selected sample with elevated weight concerns and/or behavioural symptoms.

To ensure that each trial could be included in the main analysis comparing unguided with guided self‐help, we used an algorithm to select one outcome variable from each study that best represents symptom severity: (1) global levels of eating disorder psychopathology (i.e., EDE‐Q global scores); (2) binge eating (i.e., number of episodes over the past 28 days); and (3) abstinence (complete cessation of binge eating and/or purging). We also conducted sensitivity analyses where we calculated pooled effects for each three outcomes specifically.

Table 5 presents the results of the meta‐analyses comparing unguided to guided interventions. The total pooled effect size from the 12 comparisons was a small but significant *g* = −0.26 (95% CI = −0.39, −0.13) in favour of guided interventions. There was no heterogeneity. This effect remained robust in all sensitivity analyses and when restricting the analyses to clinical samples, CBT interventions, and specific outcome variables.

**TABLE 5:** Meta‐analyses of trials comparing self‐help to guided self‐help.

Unguided versus guided self‐help	*N*_comp_	ES (95% CI)	*I* ^2^
Total effect	12	−0.26 (−0.39, −0.13)*	0%
One effect per study (smallest)	10	−0.22 (−0.36, −0.08)*	0%
One effect per study (largest)	10	−0.27 (−0.41, −0.13)*	0%
Low risk of bias trials only	5	−0.26 (−0.41, −0.11)*	0%
Trim‐and‐fill estimate	12	−0.26 (−0.38, −0.13)*	
Clinical samples only	7	−0.29 (−0.52, −0.05)*	0%
CBT interventions only	8	−0.33 (−0.51, −0.14)*	0%
Specific outcomes			
EDE(Q) global scores	8	−0.26 (−0.40, −0.12)*	0%
Binge eating	5	−0.31 (−0.51, −0.11)*	0%
Abstinence	8	0.56 (0.35, 0.88)*	0%

*Note: g* for all analyses except abstinence rates, for which OR is reported.

Abbreviation: ES = effect size.

* *p* < 0.05.

### 3.6 Dropout

Table S2 presents the meta‐analyses comparing dropout rates between self‐guided interventions and controls. The odds of dropping out were significantly higher among those allocated to the intervention relative to the control group for unselected (4 comparisons; OR = 4.26, 95% CI = 2.03, 8.95; *I* ^2^ = 90%) and pre‐selected (29 comparisons; OR = 2.10, 95% CI = 1.61, 2.74; *I* ^2^ = 61%) samples. Non‐significant differences in dropout were observed for clinical samples (16 comparisons; OR = 1.06, 95% CI = 0.77, 1.73; *I* ^2^ = 26%), even when restricting the analyses to waitlist controls (OR = 0.94, 95% CI = 0.53, 1.67, *I* ^2^ = 13%).

Table S3 presents the meta‐analyses comparing dropout rates between unguided and guided self‐help. The total pooled effect size from 11 comparisons was a non‐significant OR = 0.97 (95% CI = 0.66, 1.44), with low heterogeneity (*I* ^2^ = 39%). Non‐significant effects were also observed across sensitivity analyses and when restricting the analyses to clinical samples and CBT interventions (see Table S3).

## 4 Discussion

We located 46 trials that investigated the efficacy of self‐guided psychological interventions for the prevention or treatment of eating disorders. More than half were conducted in the past 6 years, highlighting the continued interest in these scalable forms of intervention delivery. The vast majority of trials (72%) administered a digitally‐delivered self‐guided intervention, which reflects the growing enthusiasm toward leveraging modern information technology to offer accessible mental health interventions (Torous et al. 2021). Only four trials recruited an unselected sample, demonstrating uncertainty toward the benefits of universal prevention programs delivered in a self‐guided format. More trials recruited high risk/symptomatic (*k* = 25) over clinically diagnosed samples (*k* = 17), which could reflect the prevailing view that more severe presentations warrant higher intensity treatments.

We found clear evidence that self‐guided interventions are efficacious among pre‐selected samples at high risk or who are already symptomatic. Self‐guided interventions outperformed control groups on a range of key symptom and risk measures, with effects ranging from *g* = 0.29 to 0.50. Crucially, effect sizes remained robust in various sensitivity analyses that adjusted for the influence of outliers, higher risk of bias trials, and small sample studies. Pooled effects for pre‐selected samples were considerably larger than the pooled effects observed for the small subset of trials that recruited an unselected sample. This finding is consistent with prior meta‐analyses on self‐help interventions targeting other mental health conditions (e.g., Linardon et al. 2024). Perhaps high risk samples recognise the emergence of symptoms or risk factors and are thus more motivated to engage in a self‐help programme, or have greater opportunity to show improvement in the outcomes measured than unselected samples who may be at low risk or unlikely to develop symptoms (Stice et al. 2009). Overall, the present findings demonstrate that self‐guided programs are an effective intervention format for individuals showing early signs and symptoms of eating disorders.

There was also evidence that self‐guided interventions may be effective for clinically diagnosed samples. Significant, small‐moderate effects favouring self‐guided interventions over controls were observed for reduced eating disorder psychopathology (*g* = 0.40), binge eating (*g* = 0.38), and abstinence rates (OR = 2.33). However, these results must be interpreted with a degree of caution because few studies contributed to these effects, and when restricting the analyses to specific diagnostic subgroups some of the effects became non‐significant. Specifically, sensitivity analyses on specific diagnoses show that self‐guided interventions were effective in reducing core symptoms in samples with binge‐eating disorder but not in samples with bulimia nervosa. It is well‐known that bulimia nervosa is more resistant to change than binge‐eating disorder, even for our most intensive front‐line treatments (Linardon and Wade 2018; Slade et al. 2018). This may be explained by the more complex nature of this diagnostic subtype and the different mechanisms that operate to maintain it (Cooper et al. 2004). Taken together, the available data to date indicate that self‐guided interventions may only be appropriate for binge‐eating disorder presentations, although additional trials in different diagnostic subtypes are required before strong conclusions can be made.

We identified certain factors that moderated efficacy estimates. Trials that administered a waitlist produced larger effect sizes than trials that used a more active control. This is a well‐replicated finding that has been observed in a series of meta‐analyses on psychological interventions for a range of psychiatric disorders (Cuijpers et al. 2016; Furukawa et al. 2014; Linardon 2020). Some contend that use of waitlists serves as a nocebo condition and so any effects favouring an intervention over a waitlist are likely inflated and should be interpreted as such (Furukawa et al. 2014). However, it is important to note that waitlists may be considered a suitable comparison with high external validity in light of the fact that the vast majority of people with eating disorders do not receive any form of care (Weissman and Rosselli 2017). We also found that delivery of dissonance‐based interventions produced smaller effects than other intervention types, although it is important to note that the number of studies contributing to the former subgroup was low. This finding is unexpected given that dissonance‐based programs are among the most empirically‐supported prevention programs, having been shown to reduce future onset of eating disorders in multiple trials (Stice et al. 2021). Because dissonance interventions were traditionally designed to be delivered by a trained facilitator who directs the content and structure of sessions (Becker and Stice 2017), it is possible that facilitators are an essential ingredient that accounts for much of the therapeutic effects of these programs.

A key finding from this meta‐analysis was that guided self‐help was slightly more effective than unguided self‐help. Ten trials directly compared guided to unguided self‐help, and we observed a small but significant pooled effect size of *g* = −0.26 in favour of guided programs, which remained significant in various sensitivity analyses. This finding overlaps with the pooled effect size found in a recent meta‐analysis reporting superiority of guided over unguided online programs for anxiety disorders (*g* = 0.16; 95% CI: 0.03–0.28; Oey et al. 2023). The small effect observed suggests that unguided programs may still have a meaningful public health impact, requiring far fewer resources to achieve symptom improvement. Interestingly, rate of dropout did not differ between guided and unguided programs, indicating that the added benefits of guided self‐help may not be entirely explained by higher rates of treatment engagement or completion, as has been previously suggested (Yim and Schmidt 2019). Perhaps provision of professional guidance is more effective because it keeps patients more accountable toward the change process, facilitates greater utilization of therapeutic skills in between contact sessions, or provides an opportunity for patients to check that intervention content is understood, interpreted and mastered correctly (Linardon et al. 2022).

A key question for future research involves understanding for whom guided versus unguided self‐help approaches are most appropriate. Currently, research testing moderators of response in trials comparing guided and unguided self‐help approaches is limited, with no evidence of consistent moderation effects (McClure et al. 2024). There is preliminary evidence to suggest that those with more severe symptoms at baseline experience greater benefit from guided over unguided self‐help approaches (Rohrbach et al. 2023). Therefore, it is possible that self‐guided approaches may be most suitable for those with mild‐moderate symptoms, with guided or therapist‐led approaches preserved for those with more complex psychopathology or symptom profiles, consistent with stepped‐care models. However, this finding requires replication before any firm conclusions regarding treatment matching can be made.

It is important to interpret the current findings in the context of its limitations. First, analyses were restricted to post‐intervention effects given the limited number of trials conducting longer‐term follow‐up assessments. This absence was likely due to most trials offering participants the intervention at the post‐intervention assessment after a period of waiting, precluding any comparisons at follow‐up. Second, we could not conduct any analyses on several other relevant outcomes such as future eating disorder onset because none of the trials reported outcomes like these. Incidence rates require very large samples with long follow‐up periods, which were not employed in the available trials. Third, some analyses on specific outcomes that were non‐significant may have been underpowered, given the limited number of trials reporting these outcomes. Such findings should not be taken as conclusive evidence that self‐guided interventions are not effective in targeting these specific symptoms or risk factors. Additional large‐scale trials among diverse samples are needed to confirm this.

## 5 Conclusion

We present the results of a comprehensive meta‐analysis on self‐guided psychological interventions for the prevention and treatment of eating disorders. Our findings indicate that self‐guided interventions are effective for individuals presenting with early signs and symptoms of eating disorders and are likely effective for binge‐eating disorder, with guidance further enhancing their efficacy. However, more trials sampling unselected populations and diverse diagnostic subtypes are needed before firm conclusions can be drawn about their broader applicability.

Self‐guided interventions offer a highly scalable, cost‐effective, and accessible treatment option, making them particularly valuable for individuals who may not otherwise engage with traditional care due to barriers such as cost, stigma, or service availability. Given their ability to reach a much larger population with fewer resources, these interventions could serve as an important first step in treatment, a supplement for those awaiting more intensive care, or a means of reducing public health burdens by addressing symptoms early. Their flexibility and widespread availability make them a promising avenue for increasing access to care on a larger scale.

## Ethics Statement

The authors have nothing to report.

## Conflicts of Interest

The authors declare no conflicts of interest.

## Supporting information

Supporting Information S1

## Data Availability

Data can be made available upon request.

## Endnotes

^1^
We used this definition of dropout as it is a standardized metric that was reported consistently across trials. Defining dropout in this way allowed us to pool these rates and make direct between‐study comparisons, consistent with a recent meta‐analysis on self‐guided interventions for depression (Tong et al. 2024).
