# Supplementary material for: Self‐Guided Psychological Interventions for the Treatment and Prevention of Eating Disorders: A Meta‐Analysis of Randomized Controlled Trials
Source: Eur Eat Disord Rev. 2025 Apr 14;33(5):984–94. doi: 10.1002/erv.3201 (PMC12319140; doi:10.1002/erv.3201)
Supplement: Supplementary file 1 — Supporting Information S1 [file ERV-33-984-s001.docx]

**Supplementary Materials**

**Search Terms**

1. Random* OR RCT
2. “eating disorder*” OR “bulimia nervosa” OR “anorexia nervosa” OR binge eat* OR “disordered eat*” OR “eating pathology” OR purg* OR “weight concern*” OR “shape concern*” OR “body dissatisfaction” OR “dietary restraint” OR “compulsive exercise” OR “disordered eat”
3. self*help OR self*guide* OR self*taught OR self*learn* OR self*led OR self*administer* OR self*manage* OR self*direct* OR intervention OR phone* OR smart*phone* OR telephone* OR cell*phone* OR mobile*phone* OR computer* OR multi-media OR web-based OR internet OR “online intervention” OR e-health OR unguided OR bibliotherapy

**Outcome Variable Operationalization**

- **Shape and Weight Concerns.** Included the shape and/or weight concern from the self-report or interviewer-based Eating Disorder Examination (EDE), the Weight Concern Scale, the Body Shape Questionnaire, the Body Attitudes Test, the Body Parts Scale, or the body shame scale of the Experience of Shame Scale.
- **Eating Disorder Psychopathology.** Included the global score on the EDE(Q), the Eating Attitudes Test total score, the ED-15 total score, or the Eating Disorder Diagnostic Scale composite score.
- **Binge Eating.** Included self-reported episodes of objective binge eating, items from the Questionnaire on Eating and Weight Patterns, or the total score on the Binge Eating Scale.
- **Compensatory Behaviour.** Included the frequency of self-induced vomiting, driven exercise, or laxative use episodes.
- **Dietary Restraint.** Included the restraint subscale from the EDE-Q, the Dietary Intent Scale, the Inflexible Eating Questionnaire, or the dietary restraint subscale from the Dutch Eating Behaviour Questionnaire.
- **Eating Concerns.** Included the eating concerns subscale from the EDE(Q).
- **Internalisation.** Included internalisation subscales from the Sociocultural Attitudes Towards Appearance or the Ideal-Body Stereotype Scale—Revised scale.
- **Abstinence** defined as the complete cessation of binge eating and/or purging.
- **Drop-out.** Defined as failure to complete the post-test assessment.

Additional unpublished records identified through other sources
(*N*=4)

Records identified through database searching
(*N*=5665)

Records after duplicates removed

(*N*=3775)

Records screened
(*N*=3775)

Records excluded
(*N*=3352)

Full-text articles excluded (*N*=377)

Wrong intervention (n= 320)

No relevant comparison group (n=18)

No data to calculate effects (n=3)

Wrong design (n=6)

Secondary analyses (n=19)

No relevant outcome (n=11)

Full-text articles assessed for eligibility
(*N=*423)

Studies included in meta-analysis

(*N*=46)

Figure S1: Flow-chart of Literature Search

| Table S1  Characteristics of included studies | | | | | | | | | | |
| --- | --- | --- | --- | --- | --- | --- | --- | --- | --- | --- |
|  |  | **Intervention** |  |  |  |  |  |  |  |  |
| **Study** | **Sample** | **Name (n randomized)** | **Type** | **Format** | **Module/ lesson** | **Comparison (n randomized)** | **Outcome reported** | **FU** | **Dropout** | **RoB** |
| Aardoom 2016 | Pre-selected  (≥ 52 WCS; or BMI ≤18.5, ≥ 1 weekly binge/ compensatory behavior in past month) | Featback (87) | Psycho-education + monitoring | Web | NR | Waitlist (90)  Low intensity GSH (88)  High intensity GSH (89) | ED psychopathology (EDEQ) | 8 | 27/87  18/90  18/88  18/89 | + + sr + (4) |
| Barakat 2023 | Clinical (BN, OSFED) | BEet (38) | CBT | Web | 10 | GSH CBT (37)  Waitlist (39) | OBE episodes  Vomiting episodes  Laxative episodes  Driven exercise episodes  Restraint (EDEQ)  Weight concern (EDEQ)  Shape concern (EDEQ)  ED psychopathology (EDEQ)  Eating concern (EDEQ)  Abstinence (cessation of OBE) | 12 | 12/38  13/37  7/39 | ? ? sr – (1) |
| Carter & Fairburn 1998 | Clinical (BED) | Overcoming Binge Eating (35) | CBT | Book | 6 | GSH CBT (34)  Waitlist (24) | Binge eating (EDE)  ED psychopathology (EDEQ)  Shape concern (EDEQ)  Weight concern (EDEQ)  Restraint (EDEQ)  Eating concern (EDEQ)  Abstinence (cessation of binge) | 12 | 0/35  8/34  1/24 | + + + + (4) |
| Carter 2020 | Clinical (BED) | DBT solution for emotional eating (24) | DBT | Book | NR | GSH DBT (24)  Placebo (23) | OBE episodes  ED psychopathology (EDEQ)  Abstinence (cessation of OBE) | 12 | 4/24  7/24  10/23 | ? ? + + (2) |
| Carter 2003 | Clinical (BN) | Overcoming Binge Eating (28) | CBT | Book | 6 | Placebo (28)  Waitlist (29) | Restraint (EDE)  Shape concern (EDE)  Weight concern (EDE)  Eating concern (EDE) | 8 | 5/28  7/28  8/29 | + + + + (4) |
| Chithambo 2017 | Pre-selected (WCS >34) | NR (90)  NR (88) | DBI  CBT | Web | 4 | Waitlist (93)  Waitlist (93) | ED psychopathology (EAT)  Body dissatisfaction (BSQ)  Restraint (DEBQ)  Internalization (IBSS) | 4 | 28/90  24/88  24/93 | + + sr + (4) |
| Fitzsimmons-Craft 2022 | Pre-selected (WCS > 47; or endorsement of weight being more important than most things in life or most important on the WCS; or; being very afraid or terrified of gaining three pounds on the WCS; and low risk for an ED based on not screening into one of the above categories | Tessa (352) | CBT | Chat-bot | NR | Waitlist (348) | Weight concern (WCS)  ED psychopathology (EDEQ)  Restraint (EDEQ)  Shape concern (EDEQ)  Weight concern (EDEQ)  Eating concern (EDEQ)  Internalization (SATAQ) | 12 | 191/352  158/348 | + + sr + (4) |
| Franko 2005 | Pre-selected (at risk participants categorized by the Q-EDD as “symptomatic”; no diagnosable ED, but some transient or mild symptomatic behaviors and/or behavioral risk factors). | Food, mood and attitude (120) | CBT | CD-ROM | 2 | Placebo (120) | Shape concern (EDEQ)  Weight concern (EDEQ)  Restraint (EDEQ)  ED psychopathology (EDEQ)  Internalization (SATAQ) | 12 | 4/120  5/120 | + ? sr – (2) |
| Fuller-Tyszkiewicz 2019 | Unselected | NR (202) | MBI | App | NR | Waitlist (202) | ED psychopathology (EAT) | 3 | 148/202  130/202 | ? ? sr ? (1) |
| Green 2018 | Pre-selected (clinical/subthreshold ED based on Q-EDD) | eBody Project (46) | DBI | Web | 8 | Waitlist (36) | Body dissatisfaction (BSQ)  ED psychopathology (EDEQ)  Internalization (IBSS) | NR | NR  NR | ? ? sr + (2) |
| Ghaderi 2003 | Clinical (binge spectrum EDs) | Overcoming Binge Eating (15) | CBT | Book | 6 | GSH CBT (16) | Abstinence (cessation of OBE episodes) | 16 | 6/15  7/16 | ? ? + - (2) |
| Grilo 2013 | Clinical (BED) | Overcoming Binge Eating (24) | CBT | Book | NR | Usual care (24) | ED psychopathology (EDE)  OBE episodes  Abstinence (cessation of OBE) | 16 | 0/24  0/24 | ? ? + + (2) |
| Hartmann 2024 | Clinical (BN) | Selfapy (77) | CBT | Web | 12 | Waitlist (77) | OBE episodes  Compensatory behavior episodes  ED psychopathology (EDEQ) | 12 | 17/77  8/77 | + + sr + (4) |
| Hay 2007 | Clinical (binge spectrum EDs) | NR(61) | Psychoeducation | Handouts | NR | Information resources (61) | ED psychopathology (EDEQ) | 24 | 10/61  10/61 | + + sr + (4) |
| Heron 2012 | Pre-selected (EDE-Q > 2.30 and/or BSQ > 110) | Food, mood and attitude + EMI (44)  Food, mood and attitude (44) | CBT | CD-ROM | NR | Placebo (43) | ED psychopathology (EDEQ)  Body dissatisfaction (BSQ)  Internalisation (SATAQ) | 8 | 3/44  1/44  0/43 | ? ? sr + (2) |
| Karekla 2022 | Pre-selected (> 52 WCS) | AcceptME (62) | ACT | Web | 6 | Waitlist (30) | Weight concern (WCS)  Restraint (EDEQ)  Shape concern (EDEQ)  Weight concern (EDEQ)  Eating concern (EDEQ)  ED psychopathology (EDEQ)  Body dissatisfaction (BSQ) | 4 | 32/62  2/30 | ? ? sr – (1) |
| Kass 2014 | Pre-selected (> 47 WCS) | Student Bodies (77) | CBT | Web | 8 | CBT GSH (74) | Weight concern (WCS)  OBE episodes | 8 | 18/77  22/74 | + + sr + (4) |
| Kelly 2015 | Clinical (BED) | NR (15)  NR (13) | CFT  CBT | Computer | NR | Waitlist (13)  Waitlist (13) | OBE episodes ED psychopathology (EDEQ) | 3 | 4/15  1/13 | + ? sr + (4) |
| Kelly 2018 | Clinical (AN) | NR (20) | CFT | Web | NR | Waitlist (20) | ED psychopathology (EDEQ)  Body shame (ESS) | 2 | NR  NR | + ? + ? (2) |
| Linardon 2023 | Unselected | Breaking the Diet Cycle (201) | CBT | Web | 4 | Waitlist (202) | OBE episodes  Shape concern (EDEQ)  Weight concern (EDEQ)  Restraint (IEQ)  Eating concern (EDEQ) | 4 | 111/201  53/202 | + + sr + (4) |
| Linardon 2023b | Pre-selected (>1 OBE episode per fortnight over past 3 months) | Breaking the Diet Cycle (199)  Break Binge Eating (199) | CBT  CBT | Web  App | 4  4 | Waitlist (202)  Waitlist (202) | ED psychopathology (EDEQ)  OBE episodes  Compensatory behaviors episodes  Shape concern (EDEQ)  Weight concern (EDEQ)  Dietary restraint (EDEQ)  Eating concern (EDEQ) | 4 | 106/199  47/202  73/199  47/202 | + + sr + (4) |
| Linardon 2022 | Pre-selected (presence of at least one OBE episodes in last month) | Break Binge Eating (197) | CBT | App | 4 | Waitlist (195) | ED psychopathology (EDEQ)  Shape concern (EDEQ)  Weight concern (EDEQ)  Dietary restraint (EDEQ)  OBE episodes  Compensatory behavior episodes  Eating concern (EDEQ) | 4 | 101/197  39/195 | + + sr + (4) |
| Linardon (et al 2025) | Pre-selected (>1 OBE episode per fortnight over past 3 months) | Resilience (287) | DBT | App | 5 | Waitlist (289) | OBE episodes  ED psychopathology (EDEQ)  Weight concern (EDEQ)  Shape concern (EDEQ)  Dietary restraint (EDEQ)  Compensatory behavior episodes  Eating concerns (EDEQ) | 6 | 138/287  52/289 | + + sr + (4) |
| Loeb 2000 | Clinical (BED) | Overcoming Binge Eating (20) | CBT | Book | 6 | gsh Overcoming Binge Eating (20) | OBE episodes  ED psychopathology (EDEQ)  Restraint (EDEQ)  Shape concern (EDEQ)  Weight concern (EDEQ)  Abstinence (cessation of OBE)  Eating concern (EDEQ) | 12 | NR  NR | + ? ? + (2) |
| Luo 2021 | Pre-selected (“yes” to are you currently dissatisfied with your body) | eBody Project (191) | DBI | Web | 6 | Information resources (181) | Body dissatisfaction (BSQ)  Dietary restraint (DRES)  ED psychopathology (EDDS)  Internalization (IBSS) | 6 | 5/191  2/181 | ? ? sr + (2) |
| Marx 2018 | Pre-selected (score of 3 on the item (1-5) assessing “current concerns with eating”) | Mindful Eating Coach (46) | MBI | App | NR | Waitlist (50) | Dietary restraint (DIS)  Binge eating (BES) | 3 | 2/46  0/50 | ? ? sr + (2) |
| Messer 2024 | Pre-selected (> 1 OBE episode per fortnight over past 3 months) | Mind2Body (99) | CBT | Web | 5 | Waitlist (102) | Body dissatisfaction (BSQ)  ED psychopathology (EDEQ)  Weight concern (EDEQ)  Shape concern (EDEQ)  Dietary restraint (EDEQ)  Eating concern (EDEQ)  OBE episodes  Binge eating severity (EPSI) | 4 | 36/99  28/102 | + + sr + (4) |
| Mitchell 2001 | Clinical (BN) | NR (22) | CBT | Book | NR | Pill placebo (22) | Self-induced vomiting episodes  OBE episodes | 4 | 1/22  4/22 | ? ? sr ? (2) |
| O'Brien 2023 | Pre-selected (> 1.50 EDE-Q) | Overcoming Perfectionism (27) | CBT | Web | 9 | Waitlist (25) | ED psychopathology (EDEQ) | 4 | 20/27  11/25 | ? ? sr – (1) |
| Palmer 2002 | Clinical (binge-spectrum EDs) | Overcoming Binge Eating (32) | CBT | Book | 6 | Waitlist (31)  Face to face GSH CBT (30)  Telephone GSH CBT (28) | Abstinence (cessation of OBE and purging) | 16 | 7/32  9/31  7/30  7/28 | ? ? - + (1) |
| Pennesi 2018 | Pre-selected (WCS >47) | NR (38)  NR (39) | Imagery rescripting  Cognitive dissonance | Web | NR | Mind wandering (38) | ED psychopathology (EDEQ) | 1 | 3/38  5/39  7/38 | + + sr + (4) |
| Pruessner 2024 | Clinical (BED) | Selfapy (77) | CBT | Web | 12 | Waitlist (77) | OBE episodes  ED psychopathology (EDEQ) | 12 | 17/77  10/77 | + + sr + (4) |
| Redden 2022 | Pre-selected (> 29 concerns over mistakes subscale of the FMPS) | NR (39) | Exposure (CBT) | Web | 5 | Waitlist (37) | ED psychopathology (EAT) | 2 | 3/39  2/37 | + ? sr + (3) |
| Robinson 2024 | Pre-selected ( > 46 WCS) | Focused Minds (57)  Reducing my Perfectionist Behavior (54) | CBT | Web | 8 | Waitlist (61) | ED psychopathology (ED-15) | 4 | 20/57  18/54  8/61 | + + sr + (4) |
| Rohrbach 2022 | Pre-selected (>52 WCS or BMI < 18.5, or at least weekly OBE/compensatory behavior episodes in the past 4 weeks) | Featback (88) | Psychoeducation & symptom monitoring | Web | nr | GSH Featback (90)  Waitlist (90) | ED psychopathology (EDEQ) | 8 | 12/88  22/90  21/90 | + + sr + |
| Ruwaard 2013 | Pre-selected (recurrent OBE episodes and/or inappropriate weight-control behavior, and elevated weight/shape concerns) | Overcoming Bulimia and Binge Eating (35) | CBT | Book | NR | GSH CBT (35)  Waitlist (35) | ED psychopathology (EDEQ)  Body dissatisfaction (BAT)  Abstinence (cessation of OBE)  Abstinence (cessation of purging) | 20 | 12/35  6/35  4/35 | + ? sr + (3) |
| Schmidt 2008 | Clinical (BN) | Overcoming bulimia (49) | CBT | CD-ROM | 8 | Waitlist (48) | ED psychopathology (EDE)  Restraint (EDE)  Shape concern (EDE)  Weight concern (EDE)  Abstinence (cessation of OBE and purging)  Eating concern (EDE) | 12 | 8/49  8/48 | + + + + (4) |
| Shapiro 2007 | Clinical (BED) | NR (22) | CBT | CD-ROM | NR | Waitlist (22) | OBE episodes  Binge eating severity (BES)  Abstinence (Cessation of OBE) | 10 | 7/22  2/22 | ? ? sr – (1) |
| Shu 2019 | Pre-selected (“yes” to three self-report items endorsing perfectionism) | ICBT-P (36)  ICBT-S (34) | CBT | Web | 8 | Waitlist (24) | ED psychopathology (EDEQ) | 4 | 12/36  13/34  2/24 | + + sr + (4) |
| Stice 2012 | Pre-selected (self-identified body image concerns) | eBody Project (19) | DBI | Web | 6 | Information video resource (29)  Information brochure (20) | Body dissatisfaction (BPS)  ED psychopathology (EDDS)  Dietary restraint (DES)  Internalization (IBSS) | 6 | 2/19  0/29  0/20 | ? ? + + (3) |
| Stice 2017 | Pre-selected (self-identified body dissatisfaction) | eBody Project (184) | DBI | Web | 6 | Information resources (161) | Body dissatisfaction (BPS)  ED psychopathology (EDDS)  Internalization (IBSS) | 6 | 21/184  9/161 | + ? + + (4) |
| Treasure 1994 | Clinical (BN) | NR(41) | CBT | Book | 8 | Waitlist (19) | Abstinence (cessation of OBE and purging) | 8 | 14/41  8/19 | + ? ? + (2) |
| Valentine 2018 | Pre-selected (> 24.7 on FMPS-CM and endorsement of regular exercise) | Overcoming Perfectionism (38) | CBT | Book | 8 | Waitlist (29) | ED psychopathology (EDEQ) | 8 | 9/38  3/29 | + ? sr ? (2) |
| Wilksch 2018 | Unselected | Student Bodies (190)  Media Smart (191) | CBT | Web | 9 | Information resources (194) | ED psychopathology (EDEQ)  Weight concern (WCS)  Internalization (SATAQ) | 10 | 125/190  121/191  39/194 | ? ? sr – (1) |
| Wolfe 2017 | Unselected | NR (28)  NR (35) | CBT  Gratitude | Book | NR | Placebo book (45) | Body dissatisfaction (BSQ)  ED psychopathology (EAT) | 2 | NR | ? ? sr – (2) |
| Wu 2024 | Pre-selected (self-identified as feeling dissatisfied with their appearance) | eBody Project revised (133) | DBI | Web | 3 | Information resources (99) | Internalization (SATAQ) | 1 | 48/133  22/99 | + ? sr – (2) |
| Fu = follow-up length; Nr = not reported; Gsh = guided self-help; edeq = eating disorder examination questionnaire; edds = eating disorder diagnostic test; wcs = weight concern scale; sataq = Sociocultural attitudes towards appearance questionnaire; bsq = body shape questionnaire; eat = eating attitudes test; des = dietary intent scale; obe = objective binge eating; ibss = Ideal-Body Stereotype Scale—Revised; bps = body parts scale; bat = body attitudes test; epsi = eating pathology symptoms inventory; debq = dutch eating behavior questionnaire; ieq = inflexible eating questionnaire; cbt = cognitive-behavior therapy; cft = compassion-focused therapy; act = acceptance and commitment therapy; dbi = dissonance based intervention; dbt = dialectical behavior therapy;  Rob = risk of bias; + = low risk, - = high risk; ? = unclear. In order, criteria represent sequence generation, allocation concealment, blinding of outcome or use of self-report (sr) and use of intention to treat analysis. Number in parenthesis represents how many criteria were deemed as low risk out of four. | | | | | | | | | | |

| Table S2  Meta-Analysis Comparing Dropout Rates from Self-Guided Interventions and Control Groups | | | | | | | | | | | | | |
| --- | --- | --- | --- | --- | --- | --- | --- | --- | --- | --- | --- | --- | --- |
|  | **Unselected Samples** | | |  | | **Pre-Selected Samples** | | | |  | **Clinical Samples** | | |
| **Self-Guided vs Controls** | ***N _comp_*** | ***OR* (95% CI)** | ***I^2^*** | |  | | ***N _comp_*** | ***OR* (95% CI)** | ***I^2^*** |  | ***N _comp_*** | ***OR* (95% CI)** | ***I^2^*** |
| Total effect | 4 | 4.26 (2.03, 8.95) | 90% | |  | | 29 | 2.10 (1.61, 2.74) | 61% |  | 16 | 1.06 (0.77, 1.73) | 26% |
| One effect per trial (smallest) | 3 | 3.51 (1.45, 8.50) | 91% | |  | | 23 | 2.02 (1.51, 2.70) | 57% |  | 14 | 1.07 (0.71, 1.63) | 27% |
| One effect per trial (largest) | 3 | 3.64 (1.43, 9.27) | 92% | |  | | 23 | 2.21 (1.61, 3.02) | 64% |  | 14 | 1.09 (0.71, 1.69) | 33% |
| Outliers removed | 4 | 4.26 (2.03, 8.95) | 90% | |  | | 23 | 2.07 (1.52, 2.81) | 57% |  | 16 | 1.06 (0.77, 1.73) | 26% |
| Low risk of bias trials | 1 | 4.20 (2.68, 6.59) | 0% | |  | | 16 | 1.86 (1.34, 2.59) | 74% |  | 7 | 1.19 (0.75, 1.87) | 13% |
| Trim-and-fill estimate | 4 | 4.26 (2.03, 8.95) |  | |  | | 21 | 1.88 (1.44, 2.46) |  |  | 14 | 1.14 (0.76, 1.69) |  |
|  |  |  |  | |  | |  |  |  |  |  |  |  |

| Table S3  Meta-Analysis Comparing Dropout Rates from Self-Guided and Guided Interventions | | | | |
| --- | --- | --- | --- | --- |
| **Self-Guided vs Guided** | ***N _comp_*** | ***OR* (95% CI)** | ***I^2^*** |  |
| Total effect | 11 | 0.97 (0.66, 1.44) | 39% |  |
| One effect per trial (smallest) | 9 | 0.87 (0.55, 1.38) | 42% |  |
| One effect per trial (largest) | 9 | 0.88 (0.56, 1.39) | 41% |  |
| Low risk of bias trials | 5 | 0.91 (0.46, 1.81) | 69% |  |
| Trim-and-fill estimate | 9 | 1.07 (0.71, 1.63) |  |  |
| Clinical samples only | 6 | 0.72 (0.42, 1.22) | 0% |  |
| CBT interventions only | 7 | 0.88 (0.54, 1.45) | 23% |  |

References of Included Trials

1. Aardoom, J. J., Dingemans, A. E., Spinhoven, P., van Ginkel, J. R., de Rooij, M. & van Furth, E. F. Web-based fully automated self-help with different levels of therapist support for individuals with eating disorder symptoms: A randomized controlled trial. Journal of Medical Internet Research2016; 18: 159, doi:10.2196/jmir.5709

2 Barakat, S., Burton, A. L., Cunich, M., Hay, P., Hazelton, J. L., Kim, M., . . . Miskovic-Wheatley, J. A randomised controlled trial of clinician supported vs self-help delivery of online cognitive behaviour therapy for Bulimia Nervosa. Psychiatry Research2023; 329: 115534

3 Carter, J. C. & Fairburn, C. G. Cognitive–behavioral self-help for binge eating disorder: A controlled effectiveness study. Journal of Consulting and Clinical Psychology1998; 66: 616-23, doi:10.1037/0022-006X.66.4.616

4 Carter, J. C., Kenny, T. E., Singleton, C., Van Wijk, M. & Heath, O. Dialectical behavior therapy self‐help for binge‐eating disorder: A randomized controlled study. International Journal of Eating Disorders2020; 53: 451-60

5 Carter, J. C., Olmsted, M. P., Kaplan, A. S., McCabe, R. E., Mills, J. S. & Aimé, A. Self-help for bulimia nervosa: A randomized controlled trial. The American Journal of Psychiatry2003; 160: 973-8

6 Chithambo, T. P. & Huey, S. J. Internet-delivered eating disorder prevention: a randomized controlled trial of dissonance-based and cognitive-behavioral interventions. International journal of eating disorders2017; 50: 1142‐51, doi:10.1002/eat.22762

7 Fitzsimmons-Craft, E. E., Chan, W. W., Smith, A. C., Firebaugh, M. L., Fowler, L. A., Topooco, N., . . . Jacobson, N. C. Effectiveness of a chatbot for eating disorders prevention: A randomized clinical trial. The International journal of eating disorders2022; 55: 343-53, doi:10.1002/eat.23662

8 Franko, D. L., Mintz, L. B., Villapiano, M., Green, T. C., Mainelli, D., Folensbee, L., . . . et al. Food, mood, and attitude: reducing risk for eating disorders in college women. Health Psychology2005; 24: 567‐78, doi:10.1037/0278-6133.24.6.567

9 Fuller-Tyszkiewicz, M., Richardson, B., Lewis, V., Linardon, J., Mills, J., Juknaitis, K., . . . Krug, I. A randomized trial exploring mindfulness and gratitude exercises as eHealth-based micro-interventions for improving body satisfaction. Computers in Human Behavior2019; 95: 58-65

10 Green, M. A., Kroska, A., Herrick, A., Bryant, B., Sage, E., Miles, L., . . . King, B. A preliminary trial of an online dissonance-based eating disorder intervention. Eating Behaviors2018; 31: 88-98, doi:10.1016/j.eatbeh.2018.08.007

11 Ghaderi, A. & Scott, B. Pure and guided self‐help for full and sub‐threshold bulimia nervosa and binge eating disorder. British Journal of Clinical Psychology2003; 42: 257-69

12 Grilo, C. M., White, M. A., Gueorguieva, R., Barnes, R. D. & Masheb, R. M. Self-help for binge eating disorder in primary care: A randomized controlled trial with ethnically and racially diverse obese patients. Behaviour Research and Therapy2013; 51: 855-61, doi:10.1016/j.brat.2013.10.002

13 Hartmann, S., Timm, C., Barnow, S., Rubel, J. A., Lalk, C. & Pruessner, L. Web-Based Cognitive Behavioral Treatment for Bulimia Nervosa: A Randomized Clinical Trial. JAMA network open2024; 7: e2419019, doi:10.1001/jamanetworkopen.2024.19019

14 Hay, P., Mond, J., Paxton, S., Rodgers, B., Darby, A. & Owen, C. What are the effects of providing evidence‐based information on eating disorders and their treatments? A randomized controlled trial in a symptomatic community sample. Early intervention in psychiatry2007; 1: 316-24

15 Heron, K. E. Ecological momentary intervention {emi}: Incorporating mobile technology into a disordered eating treatment program for college women 72 thesis, ProQuest Information & Learning, (2012).

16 Karekla, M., Nikolaou, P. & Merwin, R. M. Randomized Clinical Trial Evaluating AcceptME -A Digital Gamified Acceptance and Commitment Early Intervention Program for Individuals at High Risk for Eating Disorders. Journal of clinical medicine2022; 11, doi:10.3390/jcm11071775

17 Kass, A. E., Trockel, M., Safer, D. L., Sinton, M. M., Cunning, D., Rizk, M. T., . . . Taylor, C. B. Internet-based preventive intervention for reducing eating disorder risk: A randomized controlled trial comparing guided with unguided self-help. Behaviour Research and Therapy2014; 63: 90-8, doi:https://doi.org/10.1016/j.brat.2014.09.010

18 Kelly, A. C. & Carter, J. C. Self‐compassion training for binge eating disorder: A pilot randomized controlled trial. Psychology and Psychotherapy: Theory, Research and Practice2015; 88: 285-303, doi:10.1111/papt.12044

19 Kelly, A. C. & Waring, S. V. A feasibility study of a 2-week self-compassionate letter-writing intervention for nontreatment seeking individuals with typical and atypical anorexia nervosa. The International journal of eating disorders2018; 51: 1005-9, doi:10.1002/eat.22930

20 Linardon, J., Messer, M., Shatte, A., Skvarc, D., Rosato, J., Rathgen, A. & F uller-Tyszkiewicz, M. Targeting dietary restraint to reduce binge eating: a randomised controlled trial of a blended internet- and smartphone app-based intervention. Psychological Medicine2021: 1-11, doi:10.1017/S0033291721002786

21 Linardon, J., Shatte, A., McClure, Z. & Fuller-Tyszkiewicz, M. A broad v. focused digital intervention for recurrent binge eating: a randomized controlled non-inferiority trial. Psychological Medicine2022: 1-12, doi:10.1017/S0033291722001477

22 Linardon, J., Shatte, A., Rosato, J. & Fuller-Tyszkiewicz, M. Efficacy of a transdiagnostic cognitive-behavioral intervention for eating disorder psychopathology delivered through a smartphone app: a randomized controlled trial. Psychological Medicine2020: 1-12, doi:10.1017/S0033291720003426

23 Linardon, J., Anderson, C., McClure, Z., Liu, C., Messer, M., Jarman, H. & Fuller-Tyszkiewicz, M. A dialectical behaviour therapy skills training smartphone app for recurrent binge eating: A randomized clinical trial unpublished manuscript

24 Loeb, K. L., Wilson, G. T., Gilbert, J. S. & Labouvie, E. Guided and unguided self-help for binge eating. Behaviour Research and Therapy2000; 38: 259-72, doi:10.1016/S0005-7967(99)00041-8

25 Luo, Y. J., Jackson, T., Stice, E. & Chen, H. Effectiveness of an Internet Dissonance-Based Eating Disorder Prevention Intervention Among Body-Dissatisfied Young Chinese Women. Behavior Therapy2021; 52: 221-33

26 Marx, L. S. A mindful eating 'app' for non-treatment-seeking university women with eating and weight concerns 78 thesis, ProQuest Information & Learning, (2018).

27 Messer, M., Fuller-Tyszkiewicz, M., Liu, C., Anderson, C. & Linardon, J. A randomized controlled trial of an online single session intervention for body image in individuals with recurrent binge eating. International Journal of Eating Disorders2024, doi:https://doi.org/10.1002/eat.24213

28 Mitchell, J. E., Fletcher, L., Hanson, K., Mussell, M. P., Seim, H., Crosby, R. & Al-Banna, M. The relative efficacy of fluoxetine and manual-based self-help in the treatment of outpatients with bulimia nervosa. Journal of Clinical Psychopharmacology2001; 21: 298-304

29 O'Brien, A., Anderson, R., Mazzucchelli, T. G., Ure, S. & Egan, S. J. A pilot feasibility and acceptability trial of an internet indicated prevention program for perfectionism to reduce eating disorder symptoms in adolescents. Eating and weight disorders : EWD2024; 29: 27, doi:10.1007/s40519-024-01654-8

30 Palmer, R. L., Birchall, H., McGRAIN, L. & Sullivan, V. Self-help for bulimic disorders: a randomised controlled trial comparing minimal guidance with face-to-face or telephone guidance. The British Journal of Psychiatry2002; 181: 230-5

31 Pennesi, J. L. & Wade, T. D. Imagery rescripting and cognitive dissonance: A randomized controlled trial of two brief online interventions for women at risk of developing an eating disorder. The International journal of eating disorders2018; 51: 439-48, doi:10.1002/eat.22849

32 Pruessner, L., Timm, C., Barnow, S., Rubel, J. A., Lalk, C. & Hartmann, S. Effectiveness of a Web-Based Cognitive Behavioral Self-Help Intervention for Binge Eating Disorder: A Randomized Clinical Trial. JAMA network open2024; 7: e2411127, doi:10.1001/jamanetworkopen.2024.11127

33 Redden, S. A., Patel, T. A. & Cougle, J. R. Computerized treatment of perfectionism through mistake making: A preliminary study. Journal of behavior therapy and experimental psychiatry2022; 77: 101771, doi:10.1016/j.jbtep.2022.101771

34 Robinson, K., Egan, S. J., Shafran, R. & Wade, T. D. A randomised controlled evaluation of an online perfectionism intervention for people with disordered eating – How perfect does it need to be? Cognitive Behaviour Therapy2024; 53: 286-301, doi:10.1080/16506073.2024.2313739

35 Ruwaard, J., Lange, A., Broeksteeg, J., Renteria-Agirre, A., Schrieken, B., Dolan, C. V. & Emmelkamp, P. Online cognitive-behavioural treatment of bulimic symptoms: a randomized controlled trial. Clinical Psychology & Psychotherapy2013; 20: 308‐18, doi:10.1002/cpp.1767

36 Schmidt, U., Andiappan, M., Grover, M., Robinson, S., Perkins, S., Dugmore, O., . . . Williams, C. Randomised controlled trial of CD-ROM-based cognitive-behavioural self-care for bulimia nervosa. The British Journal of Psychiatry2008; 193: 493-500, doi:10.1192/bjp.bp.107.046607

37 Shapiro, J. R., Reba-Harrelson, L., Dymek-Valentine, M., Woolson, S. L., Hamer, R. M. & Bulik, C. M. Feasibility and acceptability of CD-ROM-based cognitive-behavioural treatment for binge-eating disorder. European Eating Disorders Review2007; 15: 175-84, doi:10.1002/erv.787

38 Shu, C. Y., Watson, H. J., Anderson, R. A., Wade, T. D., Kane, R. T. & Egan, S. J. A randomized controlled trial of unguided internet cognitive behaviour therapy for perfectionism in adolescents: Impact on risk for eating disorders. Behaviour Research and Therapy2019; 120: 103429

39 Stice, E., Rohde, P., Durant, S. & Shaw, H. A preliminary trial of a prototype Internet dissonance-based eating disorder prevention program for young women with body image concerns. Journal of Consulting and Clinical Psychology2012; 80: 907‐16, doi:10.1037/a0028016

40 Stice, E., Rohde, P., Shaw, H. & Gau, J. M. Clinician-led, peer-led, and internet-delivered dissonance-based eating disorder prevention programs: acute effectiveness of these delivery modalities. Journal of Consulting and Clinical Psychology2017; 85: 883‐95, doi:10.1037/ccp0000211

41 Treasure, J., Schmidt, U., Troop, N., Tiller, J., Todd, G., Keilen, M. & Dodge, E. First step in managing bulimia nervosa: controlled trial of therapeutic manual. BMJ (Clinical Research Ed.)1994; 308: 686-9

42 Valentine, E. G., Bodill, K. O., Watson, H. J., Hagger, M. S., Kane, R. T., Anderson, R. A. & Egan, S. J. A randomized controlled trial of unguided internet cognitive-behavioral treatment for perfectionism in individuals who engage in regular exercise. The International journal of eating disorders2018; 51: 984-8, doi:10.1002/eat.22888

43 Wilksch, S. M., O'Shea, A., Taylor, C. B., Wilfley, D., Jacobi, C. & Wade, T. D. Online prevention of disordered eating in at-risk young-adult women: a two-country pragmatic randomized controlled trial. Psychological Medicine2017: 1-11

44 Wolfe, W. L. & Patterson, K. Comparison of a gratitude-based and cognitive restructuring intervention for body dissatisfaction and dysfunctional eating behavior in college women. Eating disorders2017; 25: 330-44, doi:10.1080/10640266.2017.1279908

45 Wu, Y., Mulkens, S., Atkinson, M. J. & Alleva, J. M. A Brief Online Cognitive Dissonance-Based Intervention to Reduce Consideration of Cosmetic Surgery and Improve Body Image Among Chinese Women. Psychology of Women Quarterly2024; 48: 80-92, doi:10.1177/03616843231183946

46 Rohrbach, P. J., Dingemans, A. E., Spinhoven, P., Van Ginkel, J. R., Fokkema, M., Wilderjans, T. F., . . . Van Furth, E. F. (2022). Effectiveness of an online self-help program, expert-patient support, and their combination for eating disorders: Results from a randomized controlled trial. *The International Journal Of Eating Disorders, 55*, 1361-1373. doi:10.1002/eat.23785
